# Hypoglycemia during moderate intensity exercise reduces counterregulatory responses to subsequent hypoglycemia

**DOI:** 10.14814/phy2.12848

**Published:** 2016-09-04

**Authors:** W. Todd Cade, Nadia Khoury, Suzanne Nelson, Angela Shackleford, Katherine Semenkovich, Melissa J. Krauss, Ana María Arbeláez

**Affiliations:** ^1^ Program in Physical Therapy Washington University School of Medicine Saint Louis Missouri; ^2^ Department of Medicine Washington University School of Medicine Saint Louis Missouri; ^3^ Division of Biostatistics Washington University School of Medicine Saint Louis Missouri; ^4^ Department of Pediatrics Washington University School of Medicine Saint Louis Missouri

**Keywords:** Counterregulatory responses, exercise, hypoglycemia

## Abstract

Hypoglycemia, which occurs commonly during and following exercise in people with diabetes, is thought to be due to attenuated counterregulation in the setting of therapeutic insulin excess. To better understand the pathophysiology of counterregulation, we aimed to determine if dextrose administration to maintain euglycemia during moderate intensity exercise alters the attenuation of counterregulatory responses to subsequent hypoglycemia in healthy adults**.** Counterregulatory responses to hypoglycemia were assessed in 18 healthy adults after bed rest and following exercise *with* (*n* = 9*)* and *without* (*n* = 9*)* dextrose infusion. Responses were measured during a stepped euglycemic‐hypoglycemic clamp 24 h after either bed rest or two 90‐min bouts of exercise at 70% peak oxygen uptake**.** Hypoglycemia occurred during the second bout of exercise without dextrose infusion. Plasma glucagon and epinephrine responses to stepped hypoglycemia after antecedent exercise without dextrose infusion were significantly lower at the 45 mg/dL glycemic level compared to after bed rest. However, no attenuation of the counterregulatory responses to hypoglycemia was evident after antecedent exercise when dextrose was infused. This study suggests that the attenuation of the counterregulatory responses during hypoglycemia after exercise is likely due to the hypoglycemia that occurs during moderate prolonged exercise and not solely due to exercise or its intensity.

## Introduction

Counterregulatory responses to falling plasma glucose levels are normally so effective that hypoglycemia is uncommon in nondiabetic individuals (Sprague and Arbelaez 2011; Cryer 2012). However, hypoglycemia is the most common complication in people with endogenous insulin deficient diabetes and typically results from therapeutic hyperinsulinemia, compromised symptomatic responses, and impaired physiologic glucose counterregulatory defenses (Cryer 2012). During physical activity, glucose utilization is increased and energy supply in relation to the therapeutic hyperinsulinemia in patients with diabetes is frequently inadequate. Thus, episodes of hypoglycemia during and following exercise in these individuals are common and may result in seizures, coma, or death (MacDonald 1987; Chipkin et al. 2001; Ertl and Davis 2004; Tansey et al. 2006).

Controversy still exists on whether sympathoadrenal and symptomatic responses to hypoglycemia are reduced after antecedent exercise in individuals with and without diabetes. Previous studies have shown reduced sympathoadrenal and symptomatic responses to moderate hypoglycemia (~ 55 mg/dL) after moderate‐intensity exercise (50% or 70% VO_2_ max) in both healthy subjects and in those with type 1 diabetes (Schneider et al. 1991; Galassetti et al. 2001; McGregor et al. 2002; Sandoval et al. 2004, 2006). Yet research by others failed to demonstrate reduced symptomatic and autonomic responses to moderate hypoglycemia on the day following moderate‐intensity exercise (two bouts of 90 min – 50% peak oxygen consumption) in healthy subjects (Rattarasarn et al. 1994).

To better understand glucose physiology related to exercise in healthy individuals and the mechanisms that lead to attenuated counterregulatory responses to hypoglycemia after exercise in individuals with diabetes, we aimed to determine whether dextrose administration during moderate‐intensity exercise altered the attenuation of counterregulatory responses to hypoglycemia in healthy adults. Thus, we performed two sets of stepped hyperinsulinemic‐euglycemic‐hypoglycemic clamps the day after rest or after two 90‐min bouts of moderate‐intensity exercise (70% VO_2_ peak) with dextrose infusion and without dextrose infusion in healthy humans. We tested the hypothesis that dextrose infusion during exercise would prevent attenuation of the counterregulatory responses to hypoglycemia the following day.

## Materials and Methods

### Human subjects

Eighteen healthy adults participated in the study. Subjects had normal physical examinations, fasting plasma glucose concentrations, blood counts, plasma electrolytes, liver and renal function, and electrocardiogram. No subject was on any medications or had a personal or family history of diabetes, heart disease, or participated in a structured physical activity more than twice a week. All subjects gave written informed consent. The study was approved by the Washington University Human Research Protection Office and conducted at the Institute for Translational and Clinical Sciences Clinical Research Unit (CRU).

### Experimental design

**Figure 1:**
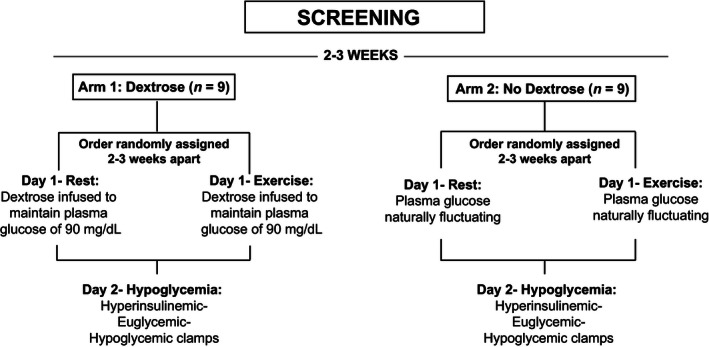
Study design. Two arm study with nine subjects in Arm 1 (with dextrose infusion) and nine subjects in Arm 2 (without dextrose infusion). In each arm, subjects exercised or rested on Day 1 in random order, and then underwent a hyperinsulinemic‐euglycemic‐hypoglycemic clamp on Day 2.

We conducted a two arm experimental study, where participants in each arm had two 2‐day experimental visits 4.7 ± 0.5 weeks apart in random order (Fig. 1). In each visit subjects underwent a euglycemic‐hypoglycemic clamp 24 h after two 90‐min cycle exercise bouts at 70% of their measured VO_2_ peak or after resting in a supine position for 6 h. The order of the visits was determined by a block randomization procedure. In Arm 1, nine subjects (4 males/5 females, 31.0 ± 10.2 years, BMI: 25.3 ± 6.2 kg/m^2^) were studied *with dextrose* infusion during the exercise and rest days; and nine other subjects (including one subject from Arm 1) (4 males/5 females, 27.8 ± 7.3 years, BMI: 22.3 ± 4.7 kg/m^2^) were studied in Arm 2 *without dextrose* infusion.

#### Screening and exercise testing visit

At least 2 weeks before the initial study, subjects performed a graded exercise test on a stationary upright cycle ergometer to determine their peak oxygen consumption (VO_2_ peak). O_2_ and CO_2_ concentrations in an expired breath were analyzed by computerized open‐circuit indirect calorimetry (ParvoMedics, Sandy, UT). The experimental work rate of 70% VO_2_ peak was chosen since it is considered moderate intensity exercise that is likely above the ventilatory threshold in healthy individuals and could be sustained for a prolonged period of time.

#### Study visits

The exercise and rest experimental visits were performed in the morning after a 10‐h overnight fast (Fig. 1). Subjects were asked to avoid any exercise and to consume their usual diet, excluding caffeine, for 3 days before each study visit. All subjects were provided a meal by the Washington University Bionutrition Unit the night before each study visit with the same caloric content based on their body weight (12 Kcal/kg, 20% protein, 25% fat, 55% carbohydrate).

##### Day 1 procedures

###### Arm 1 – With dextrose infusion

**Figure 2:**
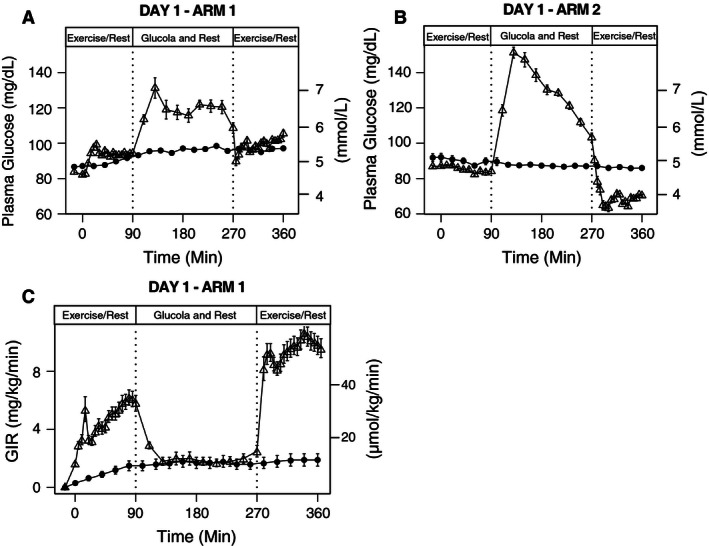
Plasma Glucose levels during exercise or rest on Day 1 studies (Exercise=▵, Rest=•). (A) Plasma glucose measurements in Arm 1 during rest or 90 min exercise bouts at 70% VO2max in the morning and afternoon¸ whereas dextrose was infused to maintain euglycemia. (B) Plasma glucose measurements in Arm 2 during rest or exercise at 70% VO2max without dextrose infusion. Mean (SE). (C) Glucose infusion rate (GIR) during exercise or rest on Day 1 of Arm 1 studies (with dextrose infusion) (Exercise=▵, Rest=•). Mean (SE).

All subjects had two intravenous lines inserted, one in the antecubital area for infusion of dextrose and one in the hand for sampling. During the exercise visit on Day 1, subjects performed two 90‐min cycle exercise bouts on a stationary cycle ergometer at ~70% VO_2_ peak from 0830 to 1000 h and from 1300 to 1430 h while having a variable amount of 20% dextrose infused to maintain euglycemia (~90 mg/dL) (Fig. 2A). Subjects rested for 180‐min between the two bouts of exercise and consumed a glucola drink containing 1.5 g carbohydrate/kg of the subject's body weight to replenish glycogen stores (Galassetti et al. 2001). VO_2_ was measured every 15 min during the exercise period to assure that subjects were exercising at the target level of VO_2_ intensity. Plasma glucose was measured approximately every 5–10 min during exercise and every 20 min during the rest periods in between the two bouts of exercise. After the second bout of exercise, subjects consumed a standardized lunch, a dinner, and a bedtime snack. All subjects remained in the CRU overnight. During the rest visit on day one, subjects were asked to rest in a supine position for 6 h and plasma glucose was measured every 20 min while an infusion of 20% dextrose was adjusted to assure that subjects were at euglycemia.

###### Arm 2 – without dextrose infusion

On Day 1 of Arm 2, subjects only had a single intravenous line inserted in the hand for sampling of plasma glucose. All subjects underwent the same experimental procedures as in Arm 1; with the exception that subjects had no dextrose infused allowing plasma glucose levels to fluctuate freely during exercise or rest (Fig. 2B).

##### Day 2 procedures for Arm 1 and 2

Two intravenous lines were inserted: one antecubital line for insulin, potassium chloride, and dextrose infusions and a contralateral hand vein for arterialized venous blood sampling (kept in a ~55°C warming plexiglas box). After 30 min of inserting the lines, potassium chloride was infused at a rate of 5 mmol/h and regular human insulin was continuously infused at 2.0 mU·kg^−1^·min^−1^ (12 pmol·kg^−1^·min^−1^) from 0 through 300 min. Dextrose (20%) was continuously infused and adjusted at variable quantities every 5 min based on plasma glucose measurements to maintain target plasma glucose concentrations during the hyperinsulinemic‐euglycemic‐hypoglycemic clamps. The target glycemic levels consisted of ~90 mg/dL (5.0 mmol/L) for 1 h, and ~55 mg/dL (3.1 mmol/L) and 45 mg/dL (2.5 mmol/L) for 2 h each. Every 30 min blood pressure, heart rate, and symptom scores were measured and an arterialized venous sample for catecholamine, insulin, glucagon, and cortisol concentrations was drawn. Electrocardiogram and vital signs were continuously monitored (Welch Allyn Propac Encore 202‐EL, Skaneateles Falls, NY) during the clamp. The hyperinsulinemic‐euglycemic‐hypoglycemic clamps were performed identically on Day 2 of the study visits in both experimental arms (Fig. 1).

Subjects scored hypoglycemic symptoms from 0 [none] to 6 [severe]. Six neurogenic (autonomic) symptoms: heart pounding, shaky/tremulousness, nervousness/anxiety (adrenergic), sweaty, hungry, and tingling (cholinergic) and six neuroglycopenic symptoms: difficulty thinking/confused, tired/drowsy, weak, warm, faint, and dizzy were scored as previously described (Towler et al. 1993). At the end of each study, insulin infusion was discontinued, a meal was served, and euglycemia was quickly restored.

### Biochemical analytical and statistical methods

#### Hormone and substrate analytes

Plasma glucose concentrations were measured with a glucose oxidase method (Yellow Springs Analyzer 2; Yellow Springs Instruments, Yellow Springs, OH). Plasma epinephrine and norepinephrine concentrations were measured with a single‐isotope derivative (radioenzymatic) method (Shah et al. 1985). Plasma insulin (Kuzuya et al. 1977), C‐peptide (Kuzuya et al. 1977), glucagon (Ensinck 1983), and cortisol (Farmer and Pierce 1974) were quantified with radioimmunoassay.

#### Statistical analysis

General linear model repeated measures analysis of variance (ANOVA) methods were implemented using SAS software version 9.3 (SAS Institute Cary, NC) to assess the difference on hypoglycemic counterregulatory responses at each glycemic level (90 mg/dL, 55 mg/dL, and 45 mg/dL) between antecedent rest and antecedent exercise conditions in each arm. Measurements at each glycemic level within each arm were compared pair‐wise and p‐values were adjusted for multiple comparisons by the Tukey post hoc method. *P* < 0.05 was considered statistical significance Data are expressed as means ± SE, except for demographic variables, which are expressed as SD.

## Results

### Day 1 exercise and rest

In experiments in Arm 1 (with dextrose infusion), subjects exercised at 69 ± 2% VO_2_ peak during the first bout of exercise and 67 ± 3% VO_2_ peak for the second bout. Comparably, in Arm 2 when dextrose was not infused during rest or cycle exercise, subjects exercised at 68 ± 2% VO_2_ peak during the first bout of exercise and 65 ± 2% VO_2_ peak for the second bout.

In Arm 1, according to our experimental design, all participants were maintained euglycemic during the first and second bout of exercise (Fig. 2A). In order to maintain euglycemia, the mean glucose infusion rate during the first bout of exercise was 4 ± 1 mg·kg^−1^·min^1^ and it increased to 10 ± 1 mg·kg^−1^·min^−1^ during the second bout of exercise (Fig. 2C). During the experiments in Arm 2, the mean plasma glucose during rest was 88 ± 2 mg/dL and the subjects remained euglycemic (85 ± 1 mg/dL) during the first bout of exercise with no dextrose infusion. However, every subject became hypoglycemic with plasma glucose below 63 mg/dL during the second bout of exercise, to a nadir of 46 mg/dL. The mean plasma glucose during this exercise period was 67 ± 3 mg/dL (Fig. 2B).

### Day 2 hyperinsulinemic‐euglycemic‐hypoglycemic clamps

**Figure 3:**
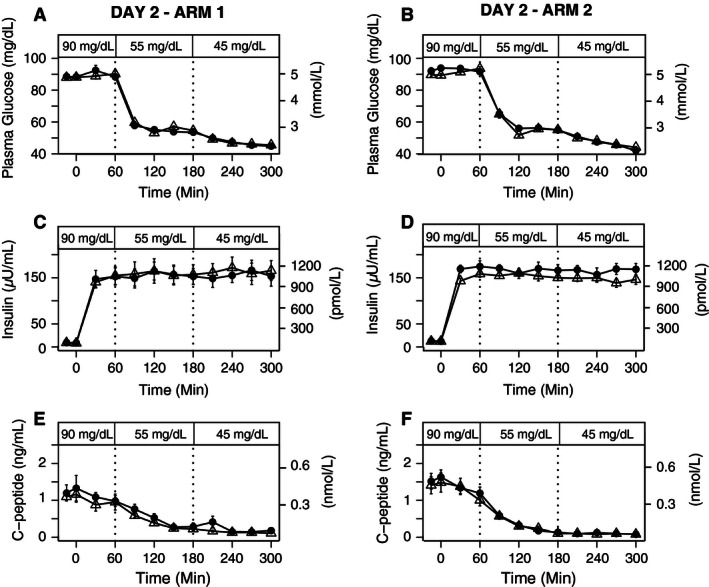
Plasma Glucose, Insulin, and C‐peptide levels during 90, 55, and 45 mg/dL stepped clamp on Day 2 after exercise or rest on Day 1 (Exercise=▵, Rest= •). (A) Plasma glucose levels during day 2 in Arm 1, (B) Plasma glucose levels during day 2 in Arm 2. (C) Insulin levels during day 2 in Arm 1 (*P* = 0.77). (D) Insulin levels during day 2 in Arm 2 (*P* = 0.84). 3E) C‐peptide levels during day 2 in Arm 1 (*P* = 0.95) and 3F) C‐peptide levels during day 2 in Arm 2 (*P* = 0.83). Mean (SE).

All subjects achieved the clamp glycemic goals set by our protocol during the hyperinsulinemic‐euglycemic‐hypoglycemic clamps on Day 2 in both arms (Fig. 3A and B). There were no significant differences in plasma glucose concentrations between Arm 1 or Arm 2 at 90, 50, or 45 mg/dL targets (Fig. 3A and B).

Plasma insulin concentrations were not significantly different between conditions or glycemic levels during the clamps following rest or exercise with dextrose infusion (*P* = 0.77), nor during the clamps following rest or exercise without dextrose infusion (*P* = 0.84) (Fig. 3C and D). Plasma C‐peptide concentrations were comparably suppressed within each glycemic level during the hyperinsulinemic clamps following exercise or rest with dextrose infusion (*P* = 0.95) in Arm 1 (Fig. 3E) and during the hyperinsulinemic clamps in Arm 2 (*P* = 0.83) without dextrose infusion (Fig. 3F).

The glucose infusion rates during the hyperinsulinemic‐hypoglycemic clamps were slightly higher on average, but not significantly different on the day after exercise compared with the day after rest in both Arm 1 (*P* = 0.95) and Arm 2 (*P* = 0.33) (Fig. 5E and F).

#### Counterregulatory responses

**Figure 4:**
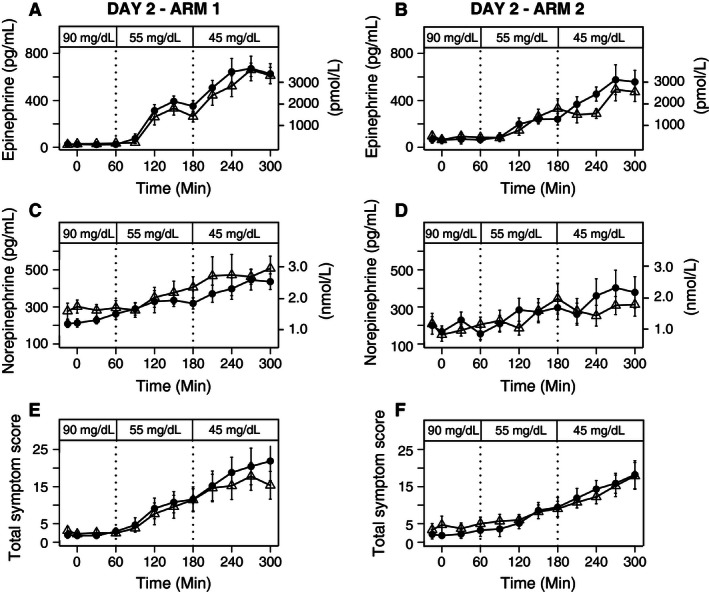
Plasma Epinephrine, Norepinephrine levels, and Total Symptom Scores during 90, 55 and 45 mg/dL stepped clamp on Day 2 after exercise or rest on Day 1 (Exercise= ▵, Rest=•). (A) Epinephrine levels during day 2 in Arm 1 (*P* = 0.45). (B) Epinephrine levels during day 2 in Arm 2 (*P* = 0.002). (C) Norepinephrine levels during day 2 in Arm 1 (*P* = 0.28), (D) Norepinephrine levels during day 2 in Arm 2 (*P* = 0.48), (E) Total Symptom Scores during day 2 in Arm 1, and (F) Total Symptom Scores during day 2 in Arm 2. Mean (SE).

On Day 2 in Arm 1, plasma epinephrine responses were similar at baseline (*rest 90 *=* *25 ± 3 pg/mL, *exercise 90 *= 29 ± 4 pg/mL) and the responses to stepped hypoglycemia (*rest 55 *=* *280 ± 31 pg/mL, *exercise 55 *=* *223 ± 30 pg/mL, *rest 45 *=* *612 ± 47 pg/mL, *exercise 45 *=* *563 ± 39 pg/mL) were not significantly different between antecedent exercise and rest (*P* = 0.45) with dextrose infusion (Fig. 4A). Contrariwise, following antecedent exercise without dextrose infusion in Arm 2, mean plasma epinephrine responses to stepped hypoglycemia were significantly lower at the 45 mg/dL after exercise than the response after rest (*exercise 45 = *383 ± 40 pg/mL*, rest 45 = *491 ± 48 pg/mL) (*P* = 0.002) (Fig. 4B). Plasma norepinephrine responses were similar at baseline and the responses to stepped hypoglycemia did not differ between exercise and rest (*P* = 0.28) (Fig. 4C) in Arm 1 (with dextrose infusion), or in Arm 2 (without dextrose infusion) (*P* = 0.48) (Fig. 4D).

**Figure 5:**
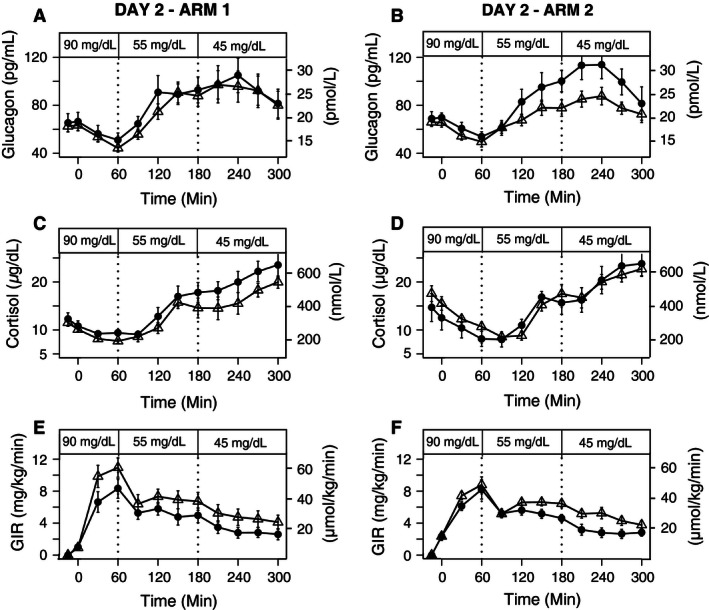
Plasma Glucagon and Cortisol levels and GIR during 90, 55, and 45 mg/dL stepped clamp on Day 2 after exercise or rest on Day 1 (Exercise=▵, Rest=•) (A) Glucagon levels during day 2 in Arm 1 (*P* = 0.72). (B) Glucagon levels during day 2 in Arm 2 (*P* = 0.002). (C). Cortisol levels during day 2 in Arm 1 (*P* = 0.18) (D) Cortisol levels during day 2 in Arm 2 (*P* = 0.18), (E) GIR during day 2 in Arm 1 (*P* = 0.95), and (F) GIR during day 2 in Arm 2 (*P* = 0.33). Mean (SE).

The mean glucagon response to stepped hypoglycemia (*rest 55 *=* *84 ± 5 pg/mL, *exercise 55 *=* *77 ± 4 pg/mL, *rest 45 *=* *94 ± 7 pg/mL, *exercise 45 *=* *91 ± 6 pg/mL) did not differ after comparing prior exercise and rest within each glycemic level (*P* = 0.72) in Arm 1 with dextrose infusion (Fig. 5A). Following antecedent exercise in Arm 2, glucagon responses to stepped hypoglycemia were attenuated only at the 45 mg/dL (*P* = 0.002), as compared to rest (*rest 55 *=* *85 ± 5 pg/mL, *exercise 55 *=* *71 ± 3 pg/mL, *rest 45 *=* *102 ± 6 pg/mL, *exercise 45 *=* *81 ± 3 pg/mL (Fig. 5B). In Arm 1, the cortisol responses to stepped hypoglycemia did not differ after comparing prior exercise and rest within each glycemic level (*P* = 0.18) (Fig. 5C). Similarly, in Arm 2 there were no significant differences (*P* = 0.18) in cortisol responses to stepped hypoglycemia (Fig. 5D).

#### Hypoglycemic symptom responses

On Day 2 in Arm 1 and Arm 2, the expected responses of total symptom scores to stepped hypoglycemia did not differ when comparing prior exercise and rest within each glycemic level (*P* = 0.19; *P* = 0.25, respectively) (Fig. 4E and F). Likewise, the neurogenic (*P* = 0.22, *P* = 0.25) and neuroglycopenic (*P* = 0.15; *P* = 0.43) symptom scores to stepped hypoglycemia did not differ after comparing prior exercise and rest in both arms (Table 1). Additionally, there was no evidence that subjects experienced different heart rates, systolic blood pressure measurements and diastolic blood pressure measurements depending on whether they received dextrose or not while exercising (*P* = 0.19, *P* = 0.65, and *P* = 0.97, respectively) (Table 1).

**Table 1:** Cardiovascular and symptomatic responses of subjects during day 2 hyperinsulinemic‐euglycemic‐hypoglycemic clamp in Arm 1 (with dextrose) and Arm 2 (without dextrose). Mean (SE) of heart rate, systolic and diastolic blood pressure, and neurogenic and neuroglycogenic symptoms

	With Dextrose Infusion on Day 1 Mean (SE)			Without Dextrose Infusion Day 1 Mean (SE)		
Glucose	90	55	45	90	55	45
Heart Rate						
Following Rest	68.5 (1.2)	79.6 (0.5)	77.6 (0.7)	67.4 (2.1)	73.4 (1.0)	74.0 (1.2)
Following Exercise	76.2 (1.9)	81.6 (1.1)	82.8 (0.6)	70.0 (0.7)	76.2 (0.8)	75.0 (0.8)
Systolic blood pressure (mmHg)						
Following Rest	115.3 (0.6)	116.8 (0.8)	114 (0.7)	119.3 (0.7)	119.2 (1.1)	123.3 (1.4)
Following Exercise	116.9 (0.4)	117.0 (1.5)	117.4 (1.4)	119.0 (0.2)	117.9 (0.8)	115.8 (0.8)
Diastolic blood pressure (mmHg)						
Following Rest	64.5 (0.6)	61.3 (0.9)	56.0 (0.6)	65.3 (0.8)	60.3 (1.8)	58.3 (0.8)
Following Exercise	64.5 (0.3)	62.2 (1.2)	57.1 (0.6)	65.1 (0.4)	60.2 (1.4)	56.7 (1.0)
Neurogenic Symptoms						
Following Rest	1.3 (0.3)	5.6 (0.9)	11.3 (1.2)	1.4 (0.4)	3.3 (0.4)	8.4 (0.7)
Following Exercise	1.3 (0.2)	4.2 (0.7)	9.4 (1.0)	1.7 (0.4)	3.4 (0.4)	7.4 (0.6)
Neuroglycopenic Symptoms						
Following Rest	1.0 (0.3)	3.5 (0.6)	7.9 (1.2)	0.9 (0.3)	3.3 (0.7)	6.7 (0.9)
Following Exercise	1.3 (0.3)	3.9 (0.8)	6.4 (1.0)	2.4 (0.6)	3.8 (0.6)	6.9 (0.9)

No secondary outcomes had significant exercise condition versus glycemic level interactions at the *α* = 0.05 level.

## Discussion

These data demonstrate that in healthy humans, the effect of antecedent moderate intensity exercise on glucose counterregulatory and symptomatic responses to subsequent hypoglycemia is modest and the observed attenuation of the hypoglycemic epinephrine and glucagon responses is likely due to the decrease in glycemia during antecedent exercise and not solely due to exercise per se or its intensity. Hypoglycemia occurring during prolonged vigorous exercise in healthy individuals is in contrast to the notion that defenses against declining plasma glucose concentrations are normally so effective that hypoglycemia is an uncommon clinical event in healthy individuals. However, it is known that a single episode of hypoglycemia, such as the one seen during exercise in our experiment, can reduce the sympathoadrenal and symptomatic responses to subsequent hypoglycemia (Davis et al. 2000; Cryer 2012), causing defective glucose counterregulation and hypoglycemia unawareness (Gold et al. 1994; Cryer 2001).

This study is the first to systematically characterize hypoglycemic responses to exercise with and without dextrose infusion. Typically insulin secretion decreases at glucose levels between 80 and 85 mg/dL (Cryer 1997). Subsequently, at glucose levels of 65–70 mg/dL, there is an increase in sympathetic neural outflow to the pancreas and the adrenal medulla (Cryer 1997; Sprague and Arbelaez 2011), which leads to an increase in the secretion of epinephrine and an increase in the secretion of glucagon. These hierarchical responses should prevent plasma glucose levels from declining to hypoglycemia in healthy individuals. However, some studies have been consistent with our findings in showing that hypoglycemia can occur in healthy subjects during moderate intensity exercise of more than 90 min duration (Felig et al. 1982; Brun et al. 2001). This is thought to be due to depletion in liver glycogen stores and the rate of glucose production failing to match the rate of glucose utilization (Sprague and Arbelaez 2011). In people with endogenous insulin deficient diabetes, hypoglycemia can occur during, shortly after, or even several hours after exercise (MacDonald 1987; Ertl and Davis 2004; Sandoval et al. 2004; Tsalikian et al. 2005), independent of the length of the exercise. However, the present data suggest that if a continuous supply of glucose is provided during the exercise, the risk of hypoglycemia during and after exercise will decrease.

It has been hypothesized that exercise induced hypoglycemia in diabetes results from the presence of relative or absolute hyperinsulinemia associated with increased insulin sensitivity (Sonnenberg et al. 1990) and blunted hypoglycemic counterregulatory responses, particularly epinephrine and glucagon responses (Schneider et al. 1991; Bottini et al. 1997; Galassetti et al. 2001). We found that in the absence of dextrose infusion during exercise at moderate intensity (70% VO_2_ peak), significantly higher glucose infusion rates were required to maintain the 45 mg/dL glycemic level during the clamp the day after exercise. Thus, in people with diabetes, strategies which aim to maintain glucose levels during exercise (e.g. decreased basal rates during exercise (Diabetes Research in Children Network Study G, et al., 2006), or maintenance of energy demands during exercise), may prevent blunting of the counterregulatory responses when hypoglycemia is subsequently experienced.

Previous research suggests that exercise at either 50% or 70% VO_2_ peak reduces epinephrine and symptomatic responses during subsequent hypoglycemia (~55 mg/dL) in both subjects that are healthy (Galassetti et al. 2001) and those with type 1 diabetes (Sandoval et al. 2004). We implemented a rigorous study design with prolonged moderate intensity exercise at 70% VO_2_ peak instead of ~50% VO_2_ max (Galassetti et al. 2001) and a markedly lower hypoglycemic clamp to 45 mg/dL. We used this more rigorous exercise and hypoglycemic paradigm because previously in a pilot study, we failed to observe an attenuation of counterregulatory responses to a plasma glucose concentration of 55 mg/dL after subjects had two 90 min bouts of exercise at 50% VO_2_ peak while having dextrose infused to maintain euglycemia the day prior compared to rest. In that experiment, we studied nine healthy individuals that met the same inclusion and exclusion criteria of this study, and found that plasma epinephrine responses during the clamp were similar at baseline and the expected incremental responses to stepped hypoglycemia did not differ after comparing prior exercise and rest. These findings are consistent with our study and others (Rattarasarn et al. 1994) in failing to demonstrate an attenuation of counterregulatory responses to hypoglycemia after exercise with dextrose infusion the previous day, suggesting that the diminished hypoglycemic counterregulatory responses, particularly epinephrine and glucagon responses, is independent of the exercise intensity.

Although not consistent with findings by Gallasetti and colleagues (Galassetti et al. 2001), the attenuation of the hypoglycemic counterregulatory responses is somewhat consistent with those of McGregor and colleagues (McGregor et al. 2002), where the impact of exercise on the counterregulatory responses to subsequent hypoglycemia after exercise was small and only noticed at the 45 mg/dL level. These data suggest that a shift of the glycemic thresholds for the epinephrine and glucagon responses to slightly lower plasma glucose concentrations occurred after antecedent hypoglycemia during exercise. In addition, as in McGregor et al. (2002), the present data show no effect of previous vigorous exercise on plasma norepinephrine or cortisol concentrations, or symptom scores as reported by Galassetti et al. (2001). The reasons for these differences are not entirely clear. However, our study was slightly different. In our study we conducted a within subject analysis, the intensity of the exercise and degree of hypoglycemia was slightly more vigorous, no insulin was infused during the exercise, and in Arm 2 there was no dextrose infused during the exercise period. Nonetheless, one would think that if the blunting of the responses was dependent on the exercise stimuli, or the degree of hypoglycemia during the clamp, we would have noticed it with our more acute hypoglycemic level of 45 mg/dL, even when dextrose was infused. Thus, we believe that the attenuation of the counterregulatory responses during hypoglycemia after exercise is likely due to the hypoglycemia that occurs during moderate prolonged exercise prior to subsequent hypoglycemia.

Our findings are limited due to the small sample size. However, the study design provides a more robust design than other studies to determine the hypoglycemic responses after subsequent exercise, since the hypoglycemic responses after exercise or rest were compared within the same subject with no dextrose or insulin infusion under identical conditions that are independent of the differences between subjects. Moreover, the analysis of the responses without dextrose infusion during exercise resembles a nonexperimental exercise paradigm that is more generalizable to normal physiology.

In summary, this study suggests that hypoglycemia occurs commonly during moderate prolonged exercise in nontrained individuals without diabetes, but hypoglycemic symptoms during exercise may sometimes be misinterpreted as fatigue and thus hypoglycemia during exercise may be underestimated. Moreover, it demonstrates that an attenuation of counterregulatory responses during hypoglycemia after exercise occurs in overnight‐fasted healthy humans, but this attenuation of the hypoglycemic counterregulatory responses after antecedent exercise appears to be due to the antecedent hypoglycemia that occurs during moderate prolonged exercise and not solely due to exercise or its intensity.

## Conflict of interest

None declared.
